# Oral Microbiota: Microbial Biomarkers of Metabolic Syndrome Independent of Host Genetic Factors

**DOI:** 10.3389/fcimb.2017.00516

**Published:** 2017-12-15

**Authors:** Jiyeon Si, Cheonghoon Lee, GwangPyo Ko

**Affiliations:** ^1^Department of Environmental Health Sciences, Graduate School of Public Health, Seoul National University, Seoul, South Korea; ^2^Institute of Health and Environment, Seoul National University, Seoul, South Korea; ^3^Center for Human and Environmental Microbiome, Seoul National University, Seoul, South Korea; ^4^N-Bio, Seoul National University, Seoul, South Korea

**Keywords:** metabolic syndrome, oral microbiome, gut microbiome, host genetics, biomarker, qPCR

## Abstract

The oral microbiota plays a critical role in both local and systemic inflammation. Metabolic syndrome (MetS) is characterized by low-grade inflammation, and many studies have been conducted on the gut microbiota from stool specimens. However, the etiological role of the oral microbiota in the development of MetS is unclear. In this study, we analyzed the oral and gut microbiome from 228 subgingival plaque and fecal samples from a Korean twin-family cohort with and without MetS. Significant differences in microbial diversity and composition were observed in both anatomical niches. However, a host genetic effect on the oral microbiota was not observed. A co-occurrence network analysis showed distinct microbiota clusters that were dependent on the MetS status. A comprehensive analysis of the oral microbiome identified *Granulicatella* and *Neisseria* as bacteria enriched in subjects with MetS and *Peptococcus* as bacteria abundant in healthy controls. Validation of the identified oral bacteria by quantitative PCR (qPCR) showed that healthy controls possessed significantly lower levels of *G*. *adiacens* (*p* = 0.023) and a higher ratio of *Peptococcus* to *Granulicatella* (*p* < 0.05) than MetS subjects. Our results support that local oral microbiota can be associated with systemic disorders. The microbial biomarkers identified in this study would aid in determination of which individuals develop chronic diseases from their MetS and contribute to strategic disease management.

## Introduction

The oral cavity serves as an initial digestive organ that breaks down carbohydrates and dietary lipids, two major energy sources for host physiology and bacterial growth. This anatomical niche has been found to have a highly diverse microbiota consisting of over 600 bacterial species (Peterson et al., 2009; Dewhirst et al., 2010). Due to its topological position, bacteria in the oral cavity are influenced by various factors such as personal hygiene (Sachdeo et al., 2008), diet (Bradshaw and Marsh, 1998), and smoke (Wu et al., 2016). Furthermore, host genetic factors may have additional effects on the oral bacteria.

Pathogenic oral bacteria are involved with periodontal diseases such as gingivitis and periodontitis (Mattila et al., 2005). In addition to local inflammation, many epidemiological studies have found an association between the presence of periodontal diseases and systemic disorders including coronary heart disease (Janket et al., 2003; Khader et al., 2004), preterm low birth (Offenbacher et al., 1998), and type 2 diabetes (Salvi et al., 2008; Chavarry et al., 2009). For the mechanism between periodontitis and cardiovascular disease, it is hypothesized that either direct invasion of oral bacteria or bacteria-mediated cytokines induce local inflammation in the cardiovascular system (Armitage, 2000; Demmer and Desvarieux, 2006; Koren et al., 2011). This is further supported by the observation that infective endocarditis is caused by *Granulicatella* and *Neisseria*, bacteria that originate from the oral cavity (Valenzuela et al., 1992; Ohara-Nemoto et al., 2005; Shailaja et al., 2013; Armingohar et al., 2014). Recently, it has also been demonstrated that administration of the oral pathobiont *Porphyromonas gingivalis* induces systemic inflammation and metabolic disorder with changes in the gut microbiota in mice (Arimatsu et al., 2014). These results potentially suggest that the oral microbiota can cause low-grade systemic inflammation in humans, leading to the development of metabolic syndrome (MetS).

MetS is characterized by visceral obesity, dyslipidemia (high levels of triglycerides [TGs] and low levels of high-density lipoprotein [HDL] cholesterol), hyperglycemia, and hypertension (Alberti et al., 2005). It is also associated with an increased risk of cardiovascular disease (CVD) and type 2 diabetes (T2D) (Grundy, 2004; Ford, 2005; Wilson et al., 2005). The oral microbiota may be a common etiological agent in these diseases; however, their role in MetS is not as clear as that of the gut microbiota (Vijay-Kumar et al., 2010; Zhang et al., 2010; Ussar et al., 2015). Assuming that the composition of the oral microbiota is associated with the health of the host, the oral cavity would be an ideal site for analyzing biomarkers because samples are comparatively easy to obtain. Previous studies reported the possibility of clinical use of oral bacteria in various systemic diseases such as pancreatic cancer, rheumatoid arthritis, and lung cancer (Farrell et al., 2012; Zhang et al., 2015), suggesting the role of oral bacteria as potential biomarkers for metabolic disorder.

With Korean twins as study subjects, we investigated the interplay between gut and oral microbiota, host genetics and MetS, and identified oral bacteria associated with the metabolic disorder. In addition, the specific oral bacteria were quantitatively evaluated using SYBR Green quantitative PCR (qPCR). The identification of oral biomarkers will lead to the development of rapid and simple diagnostic tools for metabolic diseases and contribute to strategic disease management.

## Materials and methods

### Study subjects and oral samples

A total of 228 study subjects including twins and their family members were recruited from the Healthy Twin Study as part of the Korean Genome Epidemiology Study between June 2010 and December 2012 (Sung et al., 2006). The subjects consisted of 96 monozygotic (MZ) twins, 24 dizygotic (DZ) twins, and their family members (siblings and parents, *n* = 73; Supplementary Table 1). In addition, there were 28 family members without twins and 7 unrelated subjects. We excluded subjects who took antibiotics and cold medicines (acetaminophen or ibuprofen) within 3 months of sample collection. Subgingival plaque samples were collected from the mesial sulci of the first molar using sterilized wooden toothpicks. The toothpicks were stored in normal saline (0.9% NaCl) at −70°C.

The subjects were diagnosed with MetS if they met three or more of the following criteria: waist circumference ≥90 cm in men or ≥85 cm in women, TG level ≥150 mg/dL, HDL cholesterol <40 mg/dL in men or <50 mg/dL in women, blood pressure (BP) ≥130/85 mmHg, and fasting blood sugar (FBS) ≥100 mg/dL. Waist circumference was measured horizontally at the level of the navel. Blood samples were collected from the antecubital vein for determination of TG, HDL, and FBS. TG and HDL cholesterol were measured by enzymatic and homogenous assay, respectively. FBS was measured using a hexokinase enzymatic method. BP was measured twice using a standard mercury sphygmomanometer. The study was approved by the Korea Centers for Disease Control and the Institutional Review Board of Samsung Medical Center, Busan Paik Hospital, and Seoul National University (IRB No. 144-2011-07-11).

### DNA extraction and sequencing analysis

Genomic DNA was isolated from the tip of toothpicks following the bead-beating extraction protocol (Turnbaugh et al., 2009). Briefly, the toothpick was added to 500 μL extraction buffer (200 mM NaCl, 200 mM Tris, and 20 mM EDTA; pH 8.0), 500 μL phenol:chloroform:isoamyl alcohol (25:24:1; pH 7.9) (Sigma, Steinheim, Germany), 210 μL 20% SDS, and 500 μL zirconia-silica beads (0.1 mm in diameter; Biospec Products Inc., Bartlesville, OK, USA). The mixture was homogenized using a Vortex Adaptor (Mo Bio Laboratories, Solana Beach, CA, USA) for 2 min at room temperature. DNA extraction was performed with 500 μL phenol: chloroform: isoamyl alcohol (25:24:1; pH 7.9), followed by isopropanol precipitation. The nucleic acid solutions were stored at −70°C until use. The V4 region of the 16S rRNA gene was amplified using the Illumina-adapted universal primers 515F and 806R. The samples were sequenced on the MiSeq platform using 2 × 300 bp reagent kit (Illumina, San Diego, CA, USA). Sequence data were analyzed using the QIIME software package (version 1.8.0) (Caporaso et al., 2010). Closed-reference OTU picking was performed at 97% sequence similarity based on gg_13_5 Greengenes database. Representative sequence sets were chosen using UCLUST and processed sequences were aligned using PyNAST (DeSantis et al., 2006). Taxonomy was assigned using the ribosomal database project (RDP) classifier (Cole et al., 2009) where the minimum confidence score for the taxonomy assignment to sequences was 0.8. Chimera sequences were excluded from downstream analyses prior to the generation of OTU tables using the ChimeraSlayer algorithm. OTUs were rarefied to 13,000 sequences for the oral microbiome and 7,600 sequences for the gut microbiome. Bacterial diversity within samples was assessed using the Chao1 measure (Chao, 1984), Shannon index (Shannon, 1948), and Observed Species. Statistical significance for species richness was tested using the Wilcoxon rank-sum test. Rare OTUs (singletons) were not discarded prior to the downstream analysis. Sequencing data to analyze the gut microbiome were obtained from the European Nucleotide Archive under the study accession number ERP010289.

### Co-occurrence network analysis of the microbiome in healthy controls vs. mets patients

Co-occurrence analysis of oral and gut microbiota was performed using Sparse Correlations for Compositional data (SparCC) with 500 bootstraps to estimate the *p*-value. Rarefied OTUs were collapsed at the genus level and filtered to exclude OTUs present in <50% of individuals in this study. Non-significant correlations (“two-tailed” *p* < 0.002, *q* < 0.05) were excluded and the rest of the data was plotted using Cytoscape (version 3.2.1) (Smoot et al., 2011).

### Effects of host genetics on the oral microbiome

ß-diversity analyses using weighted and unweighted UniFrac distances (Lozupone and Knight, 2005) and Bray-Curtis metrics (Bray and Curtis, 1957) were performed to compare bacterial similarity between twin pairs and unrelated subjects. The similarity of microbial abundances between MZ and DZ twin pairs was calculated by intraclass correlation using the R package irr. Heritability estimates of the oral microbiota was determined by variance component methods using Sequential Oligogenic Linkage Analysis Routines (SOLAR, version 6.6.2; West Foundation for Biomedical Research, San Antonio, TX, USA) (Almasy and Blangero, 1998). Bacterial abundances (normalized OTU counts) as quantitative traits were adjusted for age, sex, MetS, and number of bacteria per sample by fitting to a linear regression model and normalized by inverse normal transformation in R software (version 3.1.2) (R Core Team, 2017). Inverse normal transformation method matches the rank of the trait to a quantile in a normal distribution.

### Associations between oral bacteria and metabolic syndrome and validation using qPCR

Odds ratio was calculated to evaluate the association between the oral bacteria and metabolic parameters using logistic regression. A stepwise logistic regression model was constructed by a forward conditional method (SPSS, version 21; Armonk, NY, USA). Using the Student's *t*-test, bacteria selected for analysis were significantly different between MetS patients and healthy controls. The Benjamini-Hochberg FDR correction was applied where oral bacteria with *q* < 0.2 were considered significant. For the selected oral bacteria in combination, the receiver operating characteristic (ROC) curve was calculated on the cohort subjects using the predicted probabilities from logistic regression. To identify the oral biomarkers for MetS, we performed univariate LEfSe [linear discriminant analysis (LDA) coupled with effect size measurements] (Segata et al., 2011) and multivariate association tests using MaAsLin. In order to specify species of the oral biomarkers identified, oligotyping technique was performed on *Granulicatella* and *Neisseria* (Eren et al., 2013). Reads assigned to the two genera were extracted and processed separately. Entropy positions were manually chosen (-C option): 9, 10, 12, 56, 57, 58, 113, 114, 115 and 44, 57, 58, 97, 113, 114, 115, 116, 124, 135, 203, 213, 214, 229 for *Granulicatella* and *Neisseria*, respectively. The minimum substantive abundance (-M option) was set to 220 reads. Taxonomy assignment of the representative sequences of each oligotype was searched against the Human Oral Microbiome Database (HOMD) reference (version 13.2) (Chen et al., 2010) using QIIME command assign_taxonomy.py (-m blast).

Candidate bacteria were quantified by SYBR qPCR using an ABI 7300 real-time PCR system (Applied Biosystems). qPCR was performed in duplicate in a total reaction volume of 25 μL using 12.5 μL power SYBR™ Green PCR Master Mix (Applied Biosystems), 1 μL template, and 400 nM forward and reverse primers with the following cycling conditions: 95°C for 15 min, 40 cycles at 95°C for 30 s, 60°C for 1 min. Melting curve analysis was performed after amplification with default dissociation conditions: 95°C for 15 s, 60°C for 1 min, 95°C for 15 s, 60°C for 15 s. Quantification of the oral bacteria was performed using a standard curve generated from 10-fold serial dilutions of PCR fragments (*Peptococcus*) or cloned plasmid DNA (*Granulicatella* and *Neisseria*). Genomic DNA was extracted from the following type strains of oral bacteria from American Type Culture Collection (ATCC) and Korean Collection for Type Cultures (KCTC): *Peptococcus niger* (ATCC 27731), *Granulicatella adiacens* (KCTC 15209), and *Neisseria elongata* (KCTC 23361). The primer sets used are described in previous studies (Rekha et al., 2006; Farrell et al., 2012).

## Results

### Comparison of the oral and gut microbiota among patients with mets

We performed 16S rRNA gene sequencing from subgingival plaque and fecal samples from 186 healthy controls and 42 MetS patients (Supplementary Table 1). A total of 9,823,122 (mean: 43,083; range: 7,633–123,030) and 9,548,171 (mean: 41,877; range: 13,569–142,454) reads were obtained from the gut and oral microbiome, respectively. Reads were classified into species-level taxonomic bins. A total of 686 and 641 taxonomies were generated from the gut and oral microbiome, respectively. In MetS patients, 81 and 82 OTUs from the gut and oral microbiome, respectively, were found in more than 90% of the subjects (*n* = 228).

**Figure 1:**
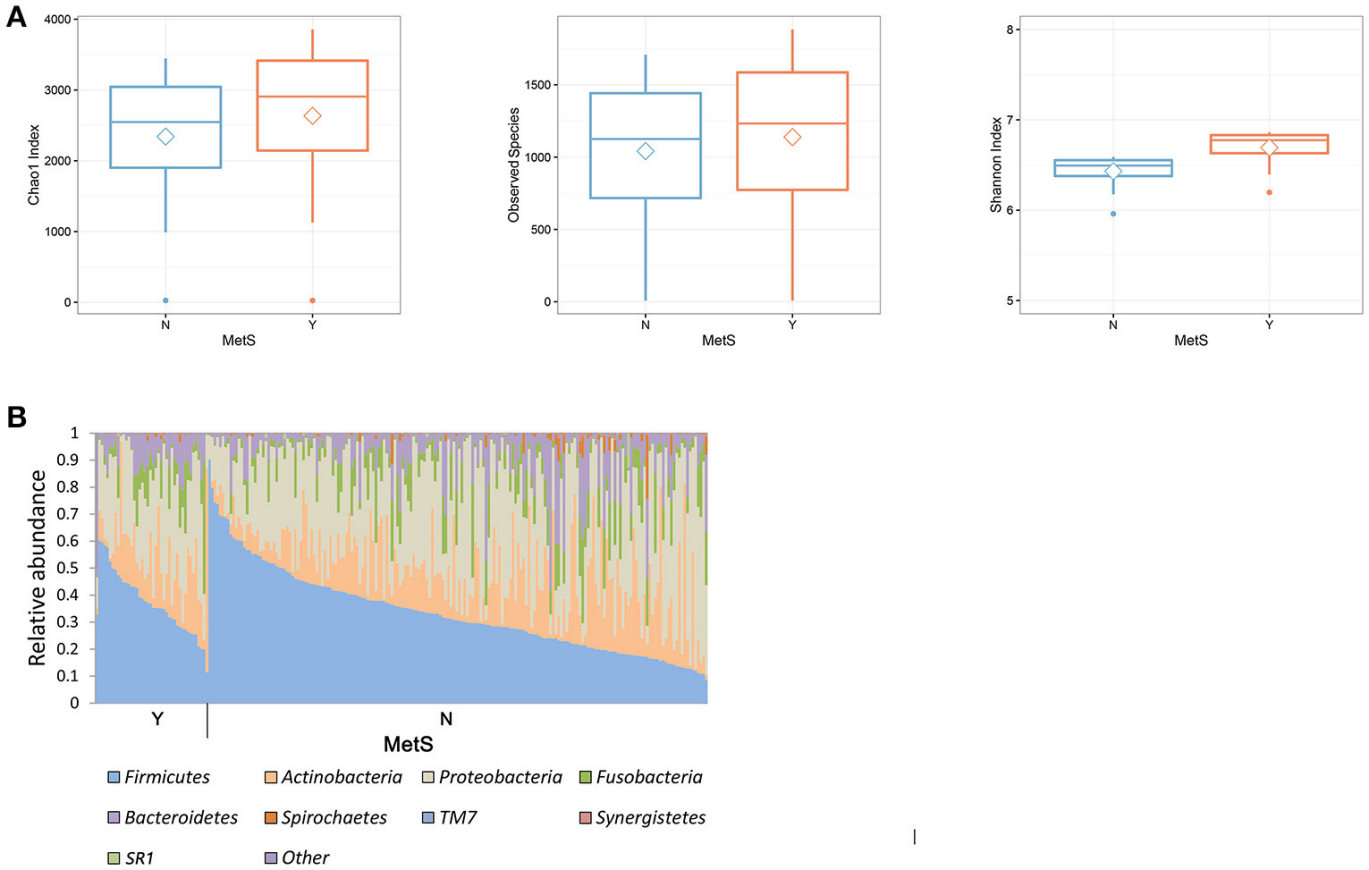
Comparison of microbial composition of oral microbiota in healthy and MetS subjects. **(A)** Boxplot of α-diversity in the oral microbiome. Boxes represent the 25th percentile, median, and 75th percentile. Whiskers and outliers represent the lowest values and the highest values of the number of OTUs (*p* < 0.05 for the Wilcoxon rank-sum test). **(B)** Microbial composition in the oral microbiome. The groups consist of 186 healthy controls and 42 MetS subjects.

A comparison of the microbial diversity between the MetS group and healthy controls showed an inverse relationship at the two body sites (Figure 1, Supplementary Figure 1). MetS patients exhibited significantly greater diversity in the oral microbiome (Wilcoxon rank-sum test: *p* = 0.001, 0.004, and 0.016 for Chao1 index, Observed Species, and Shannon index, respectively) but significantly lower diversity in the gut microbiome (Wilcoxon rank-sum test: *p* = 0.004, 0.038, and 0.017 for Chao1 index, Observed Species, and Shannon index, respectively), compared to healthy controls. In the oral microbiome, three phyla, Firmicutes (34.2%), Proteobacteria (32.3%), and Actinobacteria (16.6%), represented more than 70% of the total number of reads (Figure 1B). These percentages were similar in healthy controls (Firmicutes, 33.4%; Proteobacteria, 33.0%; and Actinobacteria, 16.4%). Compared to healthy controls, the MetS group had slightly more Firmicutes (37.9%) and slightly less Proteobacteria (29.2%). The gut microbiome was primarily composed of Bacteroidetes (51.3%) and Firmicutes (41.0%; Supplementary Figure 1B). In the MetS group, Bacteroidetes and Firmicutes accounted for 46.7 and 44.3% of the total bacteria, respectively. In healthy subjects, Bacteroidetes and Firmicutes accounted for 52.4 and 40.2% of the total bacteria, respectively. Further investigations on the oral microbiome with each metabolic parameter showed a significant increase of microbial richness within patients who met the criteria for fasting blood sugar (FBS) and TG levels (Supplementary Figure 2). With regard to HDL levels, a significant increase in microbial diversity was only observed in male subjects. A PCoA analysis of the gut and oral microbiome using different distance metrics (weighted and unweighted UniFrac distances and the Bray-Curtis distance; Supplementary Figure 3) revealed independent clusters by body site; however, the clusters could not distinguish between MetS group and healthy controls.

### Co-occurrence network analysis of the oral and gut microbiome

**Figure 2:**
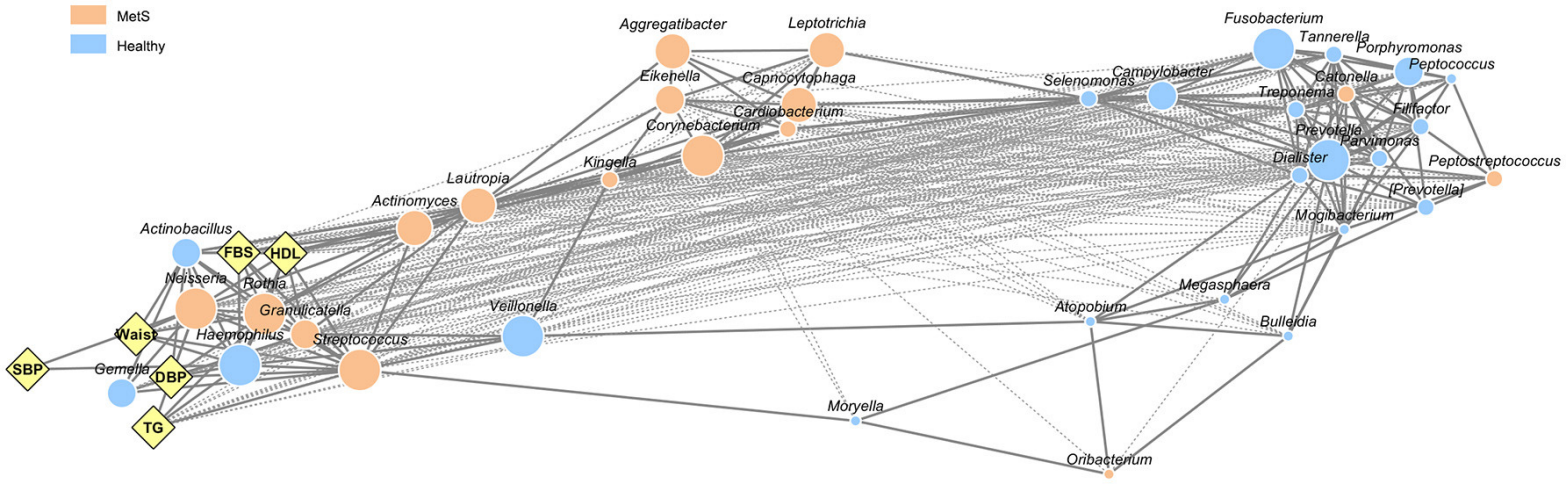
Network interaction of oral microbiome in healthy controls and MetS subjects. Edges represent significant positive (solid line) and negative (dotted line) relationships. The size of the nodes is scaled to the OTU counts of each genus. The colors reflect the bacteria enriched in healthy controls and MetS subjects as determined by Student's *t*-test (*p* < 0.05, *q* < 0.2). Diamonds indicate host parameters used to define MetS patients.

To address microbial interactions by MetS status and body site, we performed a co-occurrence network analysis from 37 oral bacteria and 33 gut bacteria found in more than 50% of the subjects. Among the oral bacteria, 296 networks including 149 positive correlations and 147 negative correlations were identified (Figure 2). Analysis of the bacterial correlation coefficients showed distinct clusters separated by MetS status. In the network of oral bacteria, *Lautropia* (enriched in MetS group), *Prevotella* (enriched in healthy controls), *Streptococcus* (enriched in the MetS group), *Dialister* (enriched in healthy controls), and *Filifactor* (enriched in healthy controls) were the top five hubs with more than 20 linkers. Host parameters showed the strongest correlation with *Streptococcus* (*r*^2^ = 0.757, 0.757, 0.711, 0.53, and 0.774 for DBP, FBS, HDL, TG, and waist, respectively) from the oral cavity. SBP had the strongest correlation with *Haemophilus* (*r*^2^ = 0.673).

The bacterial networks between the oral and the gut microbiota also showed clear distinctions between the body compartments as well as according to MetS status (Supplementary Figure 4). There were only two interactions between oral and gut bacteria in the MetS group, co-occurrence of gut *Megamonas* and oral *Actinomyces*, and co-exclusion of gut *Akkermansia* and oral *Granulicatella*.

### Influence of host genetics on human oral microbiome

**Figure 3:**
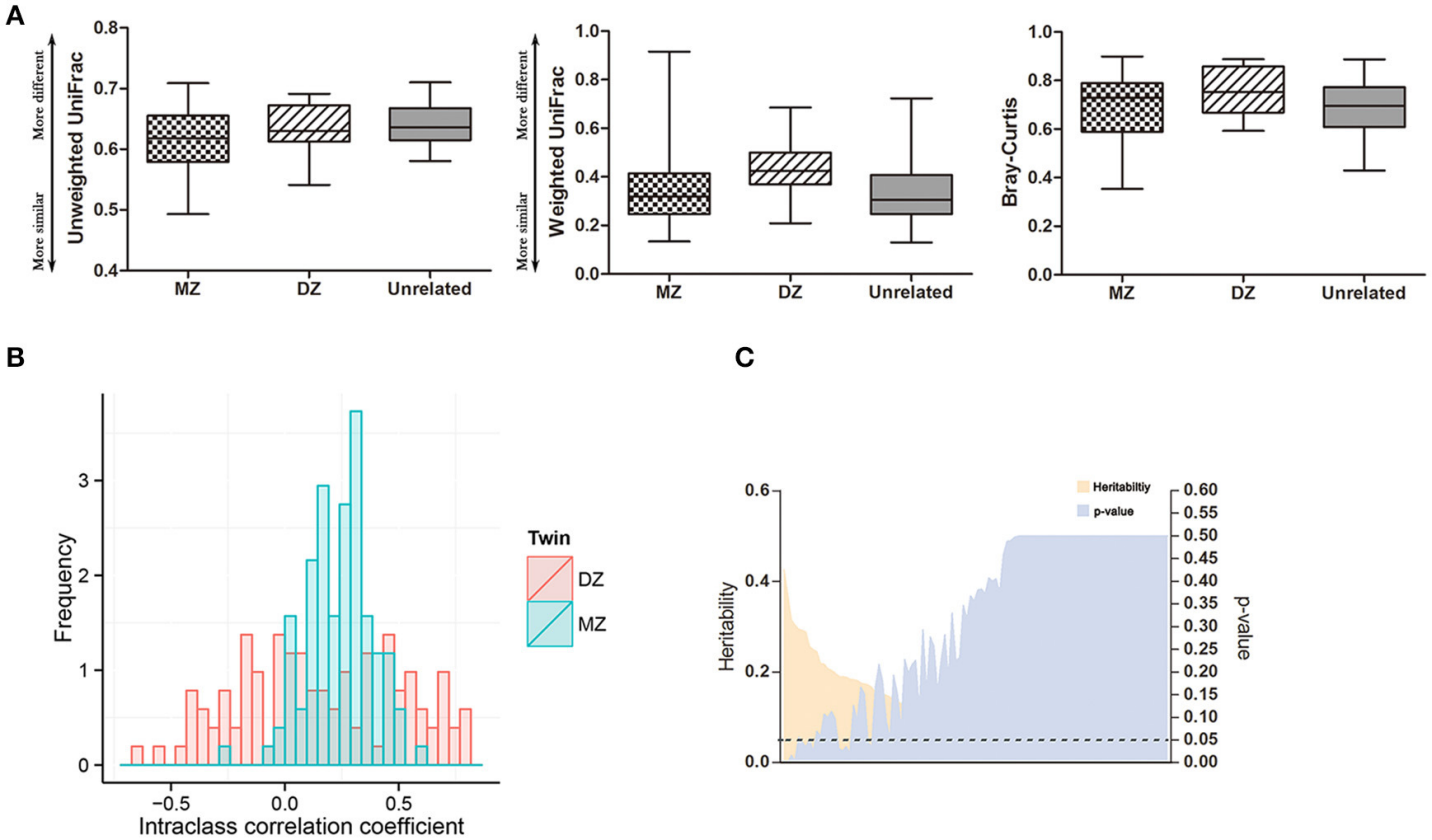
Effects of host genetics on the oral microbiome. **(A)** Comparison of β-diversity in oral microbiome under unweighted and weighted UniFrac and Bray-Curtis metrics. **(B)** Intraclass correlation coefficients (ICC) for MZ twin pairs and DZ twin pairs. **(C)** Heritability estimates of oral bacteria (orange) and corresponding multiple comparison corrected *p*-values (purple). Dashed line indicates *p* = 0.05.

To assess the effects of host genetics on the oral microbiota, we first assessed the similarities between twins and unrelated individuals (Figure 3A). The difference between monozygotic (MZ) and dizygotic (DZ) twin pairs was not statistically significant according to weighted and unweighted UniFrac and Bray-Curtis metrics (Wilcoxon rank-sum test: *p* > 0.05). The microbial abundances of twin pairs were compared using intraclass correlation coefficients (ICCs) and the results showed that host genetics do not influence the oral microbiota (Figure 3B). The group mean ICC was not significantly greater for MZ twins than DZ twins (Wilcoxon rank-sum test: *p* = 0.058). A heritability estimate of the oral microbiota showed that 13 out of 106 oral bacteria had heritable components where the ranges of the heritability estimates were between 16.6 and 42.6% (Figure 3C, Supplementary Table 2). However, the significance disappeared after adjustment of multiple comparisons. Due to non-significant effects of host genetics on the oral microbiome, we did not adjust for twins in the following analysis.

### Association of oral microbiome with mets

To determine the association of oral microbiome with MetS, we first determined 10 representative oral bacteria that were different between MetS patients and healthy controls after adjusting for age, sex, and number of bacteria (Student's *t*-test: *p* < 0.05, *q* < 0.2). *Peptococcus, Dialister, Porphyromonas*, and *Lactobacillus* were enriched in healthy controls, while *Rothia, Capnocytophaga, Granulicatella, Lautropia, Cardiobacterium*, and *Aggregatibacter* were enriched in the MetS group.

**Figure 4:**
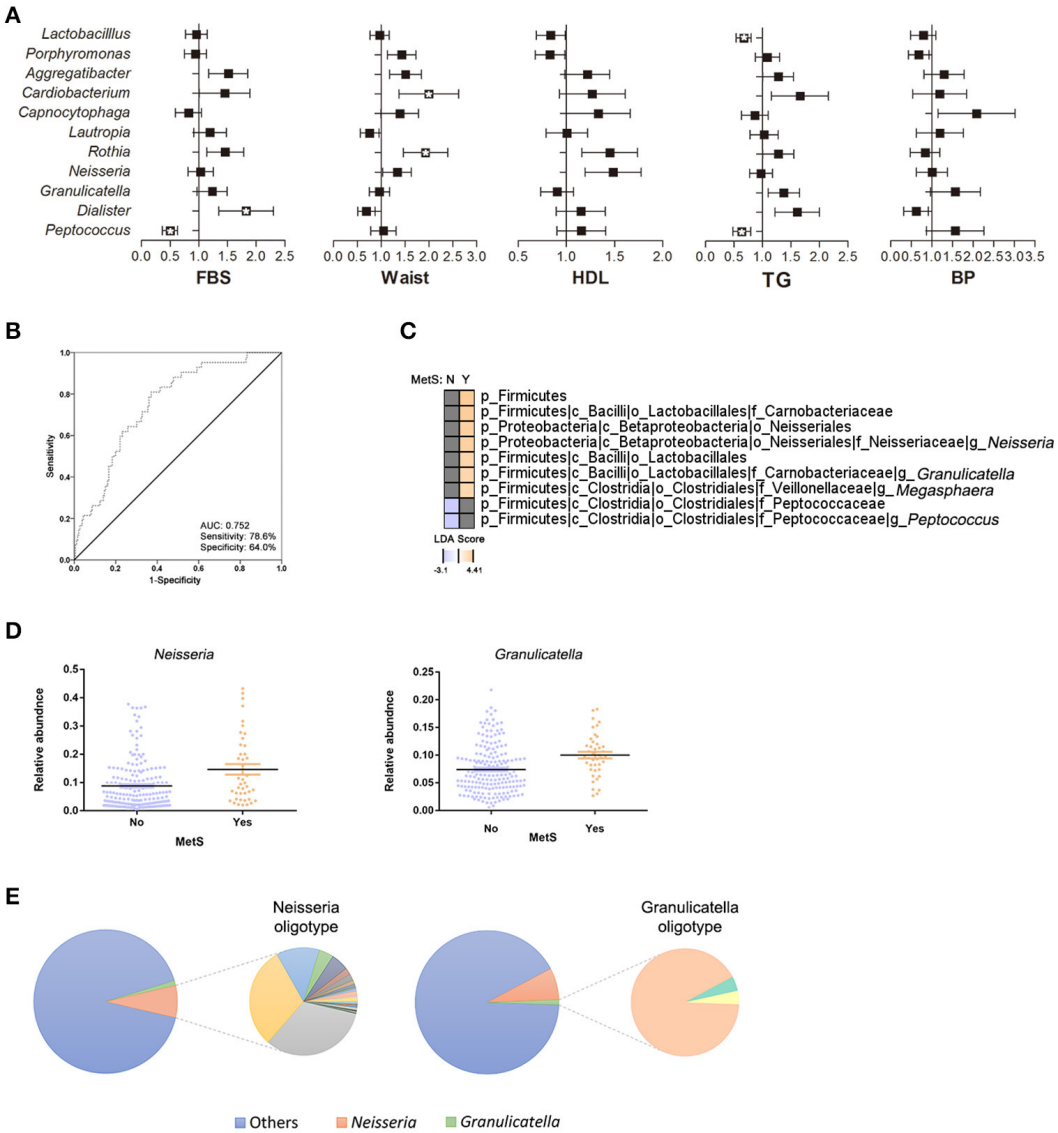
Association of oral bacteria in metabolic syndrome. **(A)** Odds ratios (ORs) for oral bacteria with risk factors of MetS. Relative abundances of bacteria are adjusted for age, sex, and number of bacteria and are transformed by inverse normalization (^*^p < 0.05, *q* < 0.2). **(B)** Receiver operating characteristic curve for the oral bacteria in ORs (95% confidence interval 0.68 to 0.83; *p* < 0.001). **(C)** Comparison of oral microbiota in healthy controls and MetS subjects using LEfSe analysis. **(D)** Significant changes in oral bacteria were analyzed by multivariate association with linear models after accounting for age, sex, and MetS status. **(E)** Oligotype distribution of genus *Neisseria* and *Granulicatella* in the entire subjects (*n* = 228): 46 and 3 oligotypes were identified for *Neisseria* and *Granulicatella*, respectively.

To further assess the impact of metabolic parameters (FBS, waist circumference, HDL, TGs, and blood pressure [BP]) on the oral microbiome, we calculated the odds ratios (ORs) for the significant oral bacteria identified between the MetS group and healthy controls (Figure 4A). Subjects who met the FBS criteria for MetS (FBS ≥ 100 mg/dL) had a significantly lower abundance of *Peptococcus* (OR 0.463, 95% CI 0.288 to 0.746, *p* = 0.002) and higher abundance of *Dialister* (OR 1.702, 95% CI 1.069 to 2.711, *p* = 0.025). The abundances of *Rothia* (OR 1.816, 95% CI 1.186 to 2.780, *p* = 0.006) and *Cardiobacterium* (OR 1.804, 95% CI 1.029 to 3.163, *p* = 0.039) were significantly greater in subjects who satisfied the waist circumference criteria. Individuals with high HDL levels had a significantly greater abundance of *Neisseria* (OR 1.428, 95% CI 1.012 to 2.015, *p* = 0.043). The abundances of *Peptococcus* (OR 0.599, 95% CI 0.389 to 0.923, *p* = 0.02) and *Lactobacillus* (OR 0.646, 95% CI 0.458 to 0.911, *p* = 0.013) were significantly lower in subjects with TG levels >150 mg/dL. Stepwise logistic regression by a forward conditional method showed a significantly greater abundance of *Granulicatella* (OR 1.519, 95% CI 1.045 to 1.519, *p* = 0.028), lower abundances of *Peptococcus* (OR 0.688, 95% CI 0.473 to 0.688, *p* = 0.028) and *Lactobacillus* (OR 0.598, 95% CI 0.411 to 0.598, *p* = 0.007) in the MetS group as compared to healthy controls (Supplementary Table 3). We evaluated the possibility that certain species of oral bacteria can be used as biomarkers for MetS. In the same cohort, the combinations of the 10 oral bacteria with ORs yielded an AUC value of 0.752 (95% CI 0.678 to 0.826, *p* < 0.001) with 78.6% sensitivity and 64.0% specificity (Figure 4B).

Next, a stepwise analysis was performed to further identify the oral bacteria enriched in MetS subjects. First, subjects were divided based on their MetS status and a univariate analysis was performed (Figure 4C). The results identified *Neisseria* (LDA score: 4.04), *Granulicatella* (LDA score: 3.49), and *Megasphaera* (LDA score: 3.47) in MetS patients and *Peptococcus* (LDA score: −3.1) in healthy controls. After adjusting for age and sex, multivariate analysis confirmed that the abundances of *Neisseria* (r-coefficient = 0.0495, *q*-value: 0.0774) and *Gragnulicatella* (r-coefficient = 0.0202, *q*-value: 0.0976) were different between the MetS group and healthy controls (Figure 4D).

Use of oligotyping method further classified genus into species (Figure 4E). In total, 46 and 3 oligotypes were identified for *Neisseria* and *Gragnulicatella*, respectively. Taxonomic classification and counts of the oligotype representative sequences are reported in Supplementary Table 4 and Supplementary Figure 5: *Gragnulicatella* oligotypes were assigned to *G. adiacens* or *G. elegans*. Highly variable *Neisseria* oligotypes were defined as *N. subflava, N. pharynges, N. elongata, N. bacilliformis*, or unknown species of genus *Neisseria*. Although not statistically significant, only 14 *Neisseria* oligotypes were more abundant in the MetS group (Wilcoxon rank-sum test: *q* > 0.1). At taxonomy level, *N. pharynges* and *N. elongata* were more abundant in the MetS group, while *N. subflava* was more abundant in the healthy controls. An additional significance analysis performed using LEfSe showed that 4 oligotypes belonging to *N. pharyngis* and 1 oligotype belonging to *N. subflava* were more abundant in the MetS group while 1 oligotype belonging to unknown species level of *Neisseria* was enriched in the control group (Supplementary Figure 6). However, the significance disappeared when the effect of age and gender was deconfounded using MaAsLin.

### qPCR validation of specific oral bacteria associated with mets

**Figure 5:**
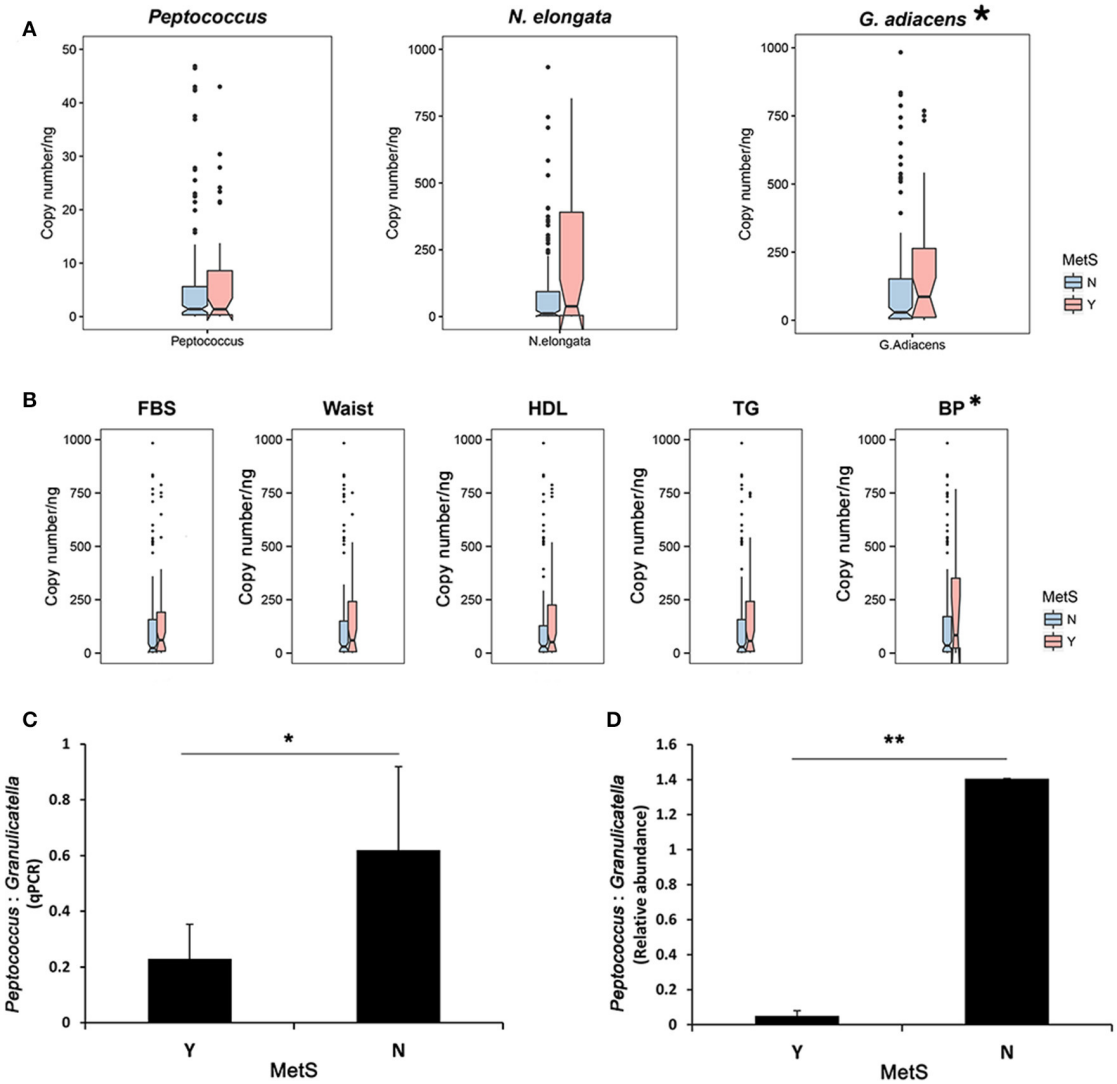
Quantification of oral bacteria associated with MetS. **(A)** Quantification of the oral biomarkers in the MetS group and healthy controls using SYBR Green qPCR. Genome copy numbers were normalized by the amount of DNA. ^*^p < 0.05 for Wilcoxon rank-sum test. **(B)** qPCR abundance of *Granulicatella adiacence* by metabolic parameters. ^*^p < 0.05 for Wilcoxon rank-sum test. *Peptococcus*:*Granulicatella* ratios in MetS subjects and healthy controls estimated from **(C)** quantification using SYBR real-time PCR and **(D)** relative abundances. Error bars, SE. ^*^p < 0.05, ^**^p < 0.005 for Wilcoxon rank-sum test.

To verify the oral bacteria identified, *N. elongata* and *G. adiacens* (MetS group) and *Peptococcus* (healthy controls) were quantified via SYBR Green qPCR. The results showed significantly increased levels of *G*. *adiacens* in subjects with MetS (Wilcoxon rank-sum test: *p* = 0.023). *N. elongata* showed a tendency toward significant increase in the MetS group (*p* = 0.074), however, *Peptococcus* was not significantly different between the two groups (Figure 5A). Next, metabolic parameters associated with specific oral bacteria were analyzed. Subjects whose BP met the criteria for MetS had significantly greater levels of *G*. *adiacens* (Figure 5B; Wilcoxon rank-sum test: *p* = 0.048). The ratio of *Peptococcus* to *G*. *adiacens* was significantly lower in MetS patients (Figure 5C, Student's *t*-test: *p* = 0.023), which confirmed the results from the microbiome analysis (Figure 5D, Student's *t*-test: *p* = 0.002).

## Discussion

Our results demonstrate that local oral bacteria can be used as biomarkers for systemic disorders such as MetS. To investigate the dynamics of the oral microbiome associated with MetS and identify oral bacterial biomarkers, we performed various statistical analyses and partially confirmed the sequencing results through qPCR. Analysis of microbial diversity showed opposite results for the oral and gut microbiome: individuals with the MetS had reduced diversity in the gut, while it was increased in the oral cavity. This corresponds with results from a previous study that found reduced gut microbial diversity in obese twins (Turnbaugh et al., 2009). Excessive nutrient levels in the obese subjects were suspected to result in over-blooming of Firmicutes, and subsequent overwhelming of other gut microbes. Blood lipids, which are strong risk factors for CVD, were also reported to be associated with microbial diversity (Fu et al., 2015): in a large Netherlands cohort, the diversity of the gut microbiota was reduced in subjects with higher BMI, higher TG, but lower HDL. In light of the fundamental roles of the gut microbiota on human health, reduction of the bacterial species in the gut implies functional and metabolic defects in hosts. In contrast, reduced diversity of the oral microbiota was reported to indicate good oral health including less number of decayed teeth, periodontal pockets, bleeding on probing, and non-smoking history (Takeshita et al., 2016). Local inflammatory responses caused by dental implants and periodontitis were shown to increase the microbial diversity (Camelo-Castillo et al., 2015; Zheng et al., 2015). In addition to the local inflammation, a higher diversity and bacterial load have been reported in patients with vascular disease (Armingohar et al., 2014). The authors of the study hypothesized that the surplus bacteria in the oral cavity acted as a gateway for the entry of pathogenic bacteria, leading to cardiovascular disease. Indeed, a recent study reported that oral administration of the periodontal pathogen, *P*. *gingivalis*, increased levels of plasma endotoxin and decreased gene expression of a tight junction protein in the small intestine in mice (Arimatsu et al., 2014). Given that patients with obesity, cardiovascular disease, and MetS all have low-grade inflammation, these results strongly suggest that oral bacteria can lead to systemic disorders.

Analyses of host genetic factors showed little or no influence of host genetics on the oral microbiome. These results are consistent with a previous study on the salivary microbiome of a small sample of twins (54 MZ and 36 DZ twins) at an early age (age 12–24) (Stahringer et al., 2012). Despite differences in the sampling site (subgingival plaque) and the age of the patients compared to our study, effects of host genetics did not affect the oral microbial community. Hence, we suspect that other environmental factors have a much greater influence on the structure of the human oral microbiome. Indeed, a recent large cohort study confirmed that smoking is an environmental factor that can influence the oral microbiome (Wu et al., 2016). Further study is warranted to investigate how environmental factors such as diet, medication, and personal hygiene influence the microbial structure and the oral health.

Metabolic parameters used for the diagnosis were found to independently influence the oral microbiome. *Peptococcus*, which was significantly associated with healthy levels of FBS and TG in the present study, has been shown to reflect healthy dental conditions as it is found at lower abundances in subjects with caries, smokers, and esophageal carcinoma (Bizzarro et al., 2013; Jiang et al., 2014; Chen et al., 2015). A low HDL level, a risk factor for cardiovascular disease (Assmann and Gotto, 2004), has been shown to be significantly associated with *Neisseria*. The causal role of *Neisseria* in various cardiovascular diseases such as acute heart failure and endocarditis supports their association with low HDL (Benes et al., 2003; Taldir et al., 2013). Through multiple analytical steps and taking account of age and gender, we found a significant association between *G*. *adiacens* and MetS, more specifically, BP. *Granulicatella*, originally known as a nutritional variant of *Streptococcus*, has been commonly reported to cause infective endocarditis (Ohara-Nemoto et al., 2005; Shailaja et al., 2013). Comparatively strong infectivity of the oral bacteria has been attributed to their ability of fibrinonectin binding to the cardial valvular tissue (Okada et al., 2000; Dowd et al., 2008), which could possibly influence BP by damaging the control valve. Additionally, identification of these bacteria primarily in blood and endovascular infection (Chang et al., 2008) suggests their systemic effects. The results of *Neisseria* not confirmed by qPCR is possibly due to the oligotypes classified into the same taxonomy showing different behavior in the MetS group. Although it was not statistically significant, test of *N. pharyngis* as another potential biomarker would be interesting given that it was highly abundant in the MetS group (Supplementary Figure 5).

Pathophysiologic nature of MetS arises from its association with other chronic disease such as CVD and T2D. These life course diseases have a number of risk factors such as family history, smoking, diet, stress, high blood cholesterol and pressure, which makes it difficult to treat the diseases. Yet, oral microbial biomarkers identified from MetS patients are advantageous as they can aid in determination of which individuals develop CVD and T2D from their MetS. Currently, the diagnosis of MetS requires three out of the following metabolic risk factors: greater waist circumference, elevated levels of TG, reduced HDL cholesterol, elevated BP, and elevated FBS (Alberti et al., 2009). Oral microbial biomarkers can cover the overall disease state and provides information of patients who have clinically similar background. Therefore, these biomarkers can potentially contribute to strategic disease management along with the current diagnostic parameters.

A limitation of this study lies in that the oral biomarkers were not tested in other populations. It has been reported that classification of the oral microbiome according to ethnicity is highly feasible (Mason et al., 2013). Although we were unable to confirm genetic effects on the oral microbiota, few oral bacteria were found to have a heritable component, which could possibly drive ethnic divergence in the oral cavity. However, the oral biomarkers identified in this study were least influenced by host genetics. Furthermore, these biomarkers have been reported in numerous case-report studies in Western countries, indicating their potential value for clinical use.

Our comprehensive analysis of the oral microbiome in parallel with the gut microbiome supports the notion that metabolic disease can influence the non-gut human microbiome. Using rigorous analytical methods and a large sample size from a twin-family cohort, we demonstrated the use of local oral bacteria as potential biomarkers for systemic disease. Microbial biomarkers hold great potential as noninvasive diagnostic measures of systemic disease. Further studies on subpopulations and studies on bacterial populations after disease treatment are warranted to test the feasibility of the oral microbial biomarkers. This way, it will lead to the development of therapeutic biological markers for metabolic diseases.

## Data availability

The sequence data have been submitted to the EMBL databases under accession number ERP014487 (http://www.ebi.ac.uk/ena).

## Author contributions

JS and CL performed the experiments. JS, CL, and GK conceived the study. JS and GK analyzed the data and prepared the manuscript.

### Conflict of interest statement

The authors declare that the research was conducted in the absence of any commercial or financial relationships that could be construed as a potential conflict of interest.
