# Supplementary material for: Oral Microbiota: Microbial Biomarkers of Metabolic Syndrome Independent of Host Genetic Factors
Source: Front Cell Infect Microbiol. 2017 Dec 15;7:516. doi: 10.3389/fcimb.2017.00516 (PMC5736563; doi:10.3389/fcimb.2017.00516)
Supplement: Supplementary file 5 [file DataSheet1.docx]

Supplementary Material

Oral microbiota: Microbial biomarkers of metabolic syndrome independent of host genetic factors

Jiyeon Si, Cheonghoon Lee, GwangPyo Ko ^*^

*** Correspondence:** Dr. GwangPyo Ko, E-mail: [gko@snu.ac.kr](mailto:gko@snu.ac.kr)

**
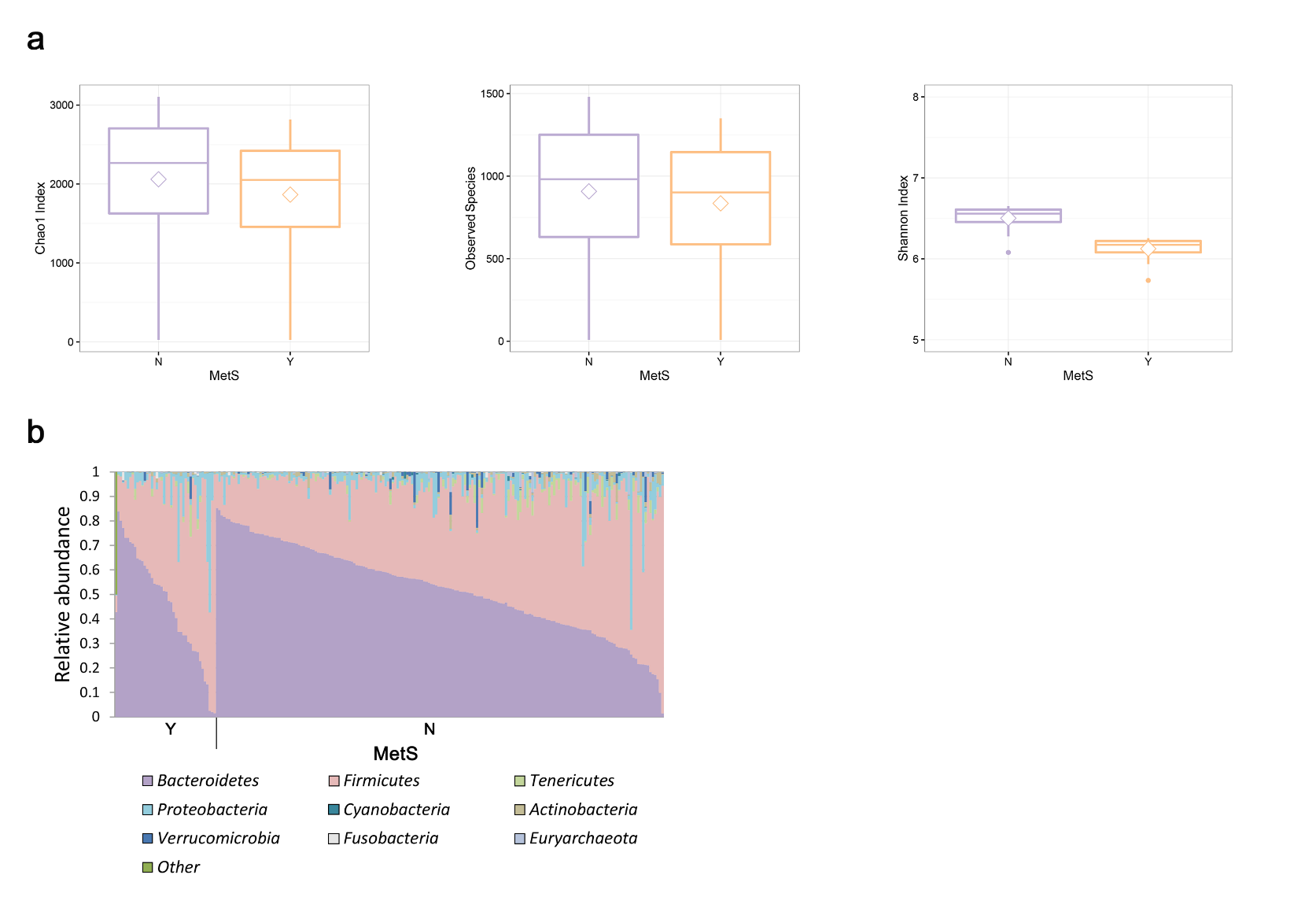
**

**Supplementary Figure 1.** Comparison of microbial composition of gut microbiota in healthy and MetS subjects. **(a)** Boxplot of α-diversity in the gut microbiome. Boxes represent the 25^th^ percentile, median, and 75^th^ percentile. Whiskers and outliers represent the lowest values and the highest values of the number of OTUs (*p* < 0.05 for the Wilcoxon rank-sum test). **(b)** Microbial composition in the gut microbiome. The groups consist of 186 healthy controls and 42 MetS subjects.


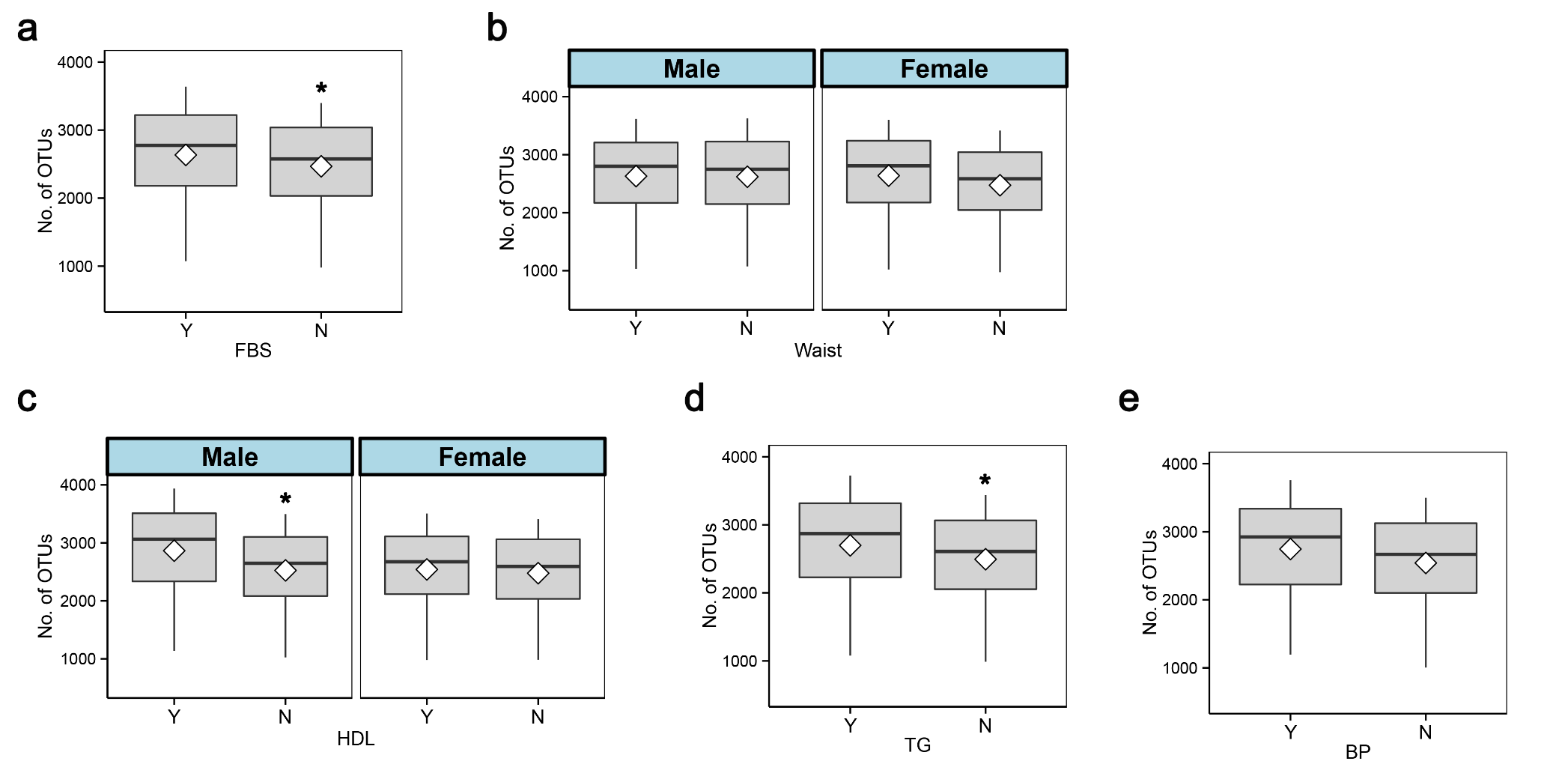


**Supplementary Figure 2. Comparison of oral microbial diversity by metabolic parameters. (a)** FBS, **(b)** waist, **(c)** HDL, **(d)** TG, **(e)** BP. Y indicates that the group met the criteria for MetS in each parameter. **p* < 0.05 for Wilcoxon rank-sum test.


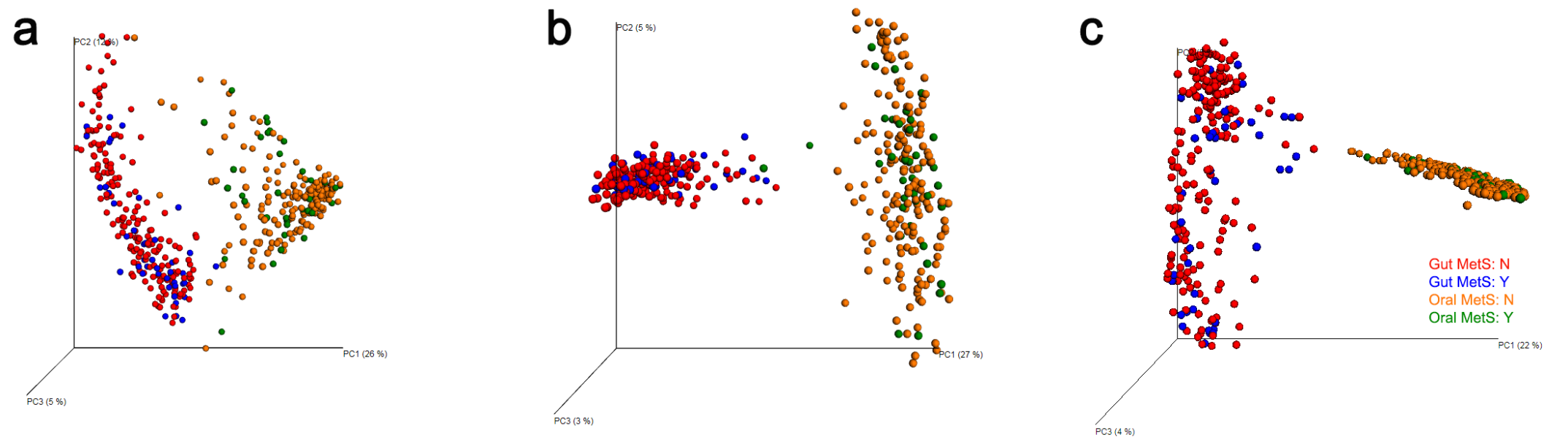


**Supplementary Figure 3. PCoA plots of oral and gut microbiome between healthy controls and MetS subjects.** Plots were generated from **(a)** weighted UniFrac distance, **(b)** unweighted UniFrac distance, and **(c)** Bray-Curtis distance.


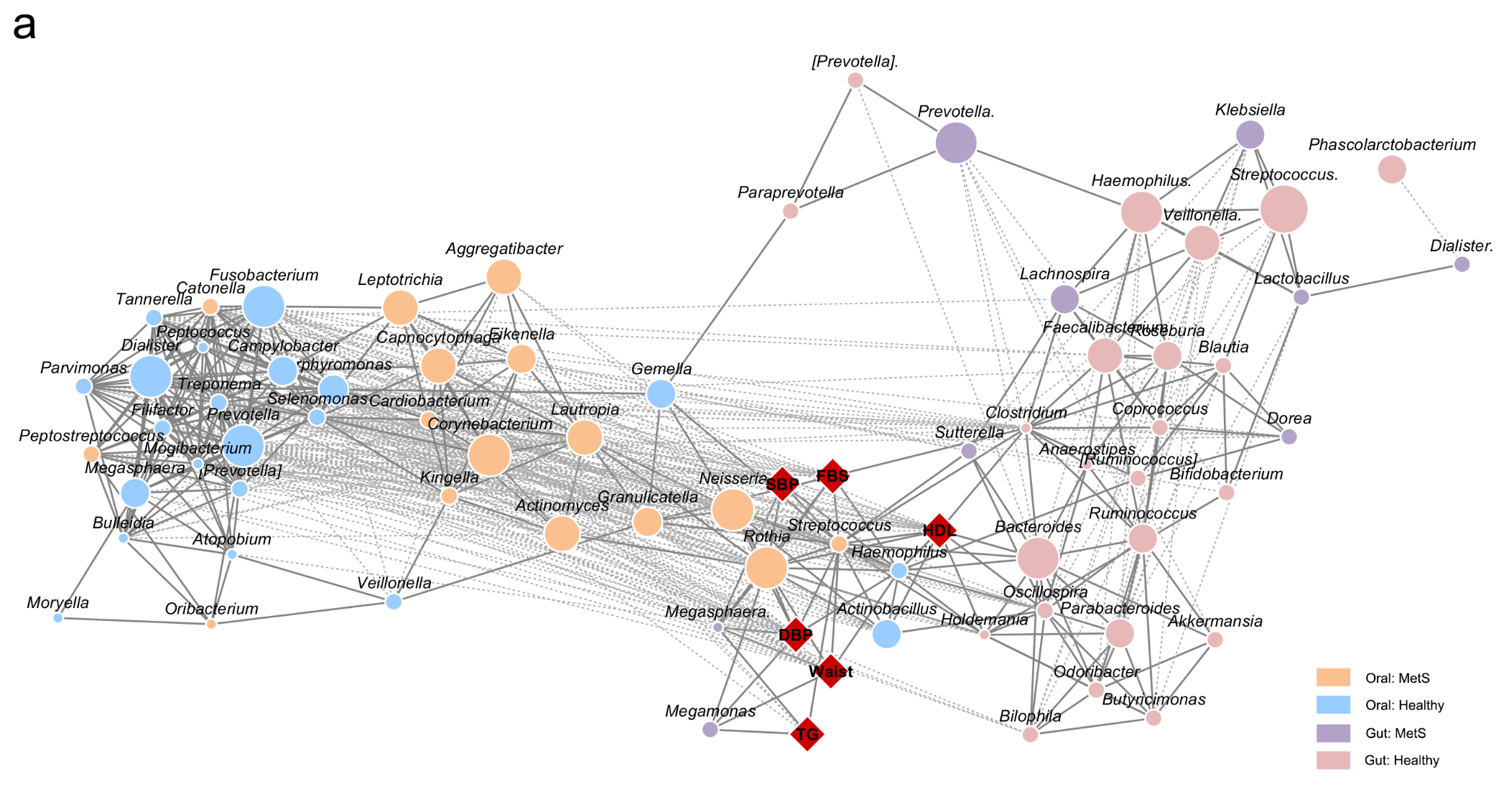


**Supplementary Figure 4.** **Network interaction of** **oral and gut microbiome.** **(a)** Microbial co-occurrence and co-exclusion relationships in healthy controls and MetS subjects and **(b)** in MetS subjects only. Edges represent significant positive (solid line) and negative (dotted line) relationships. The size of the nodes is scaled to the relative abundance of each genus.


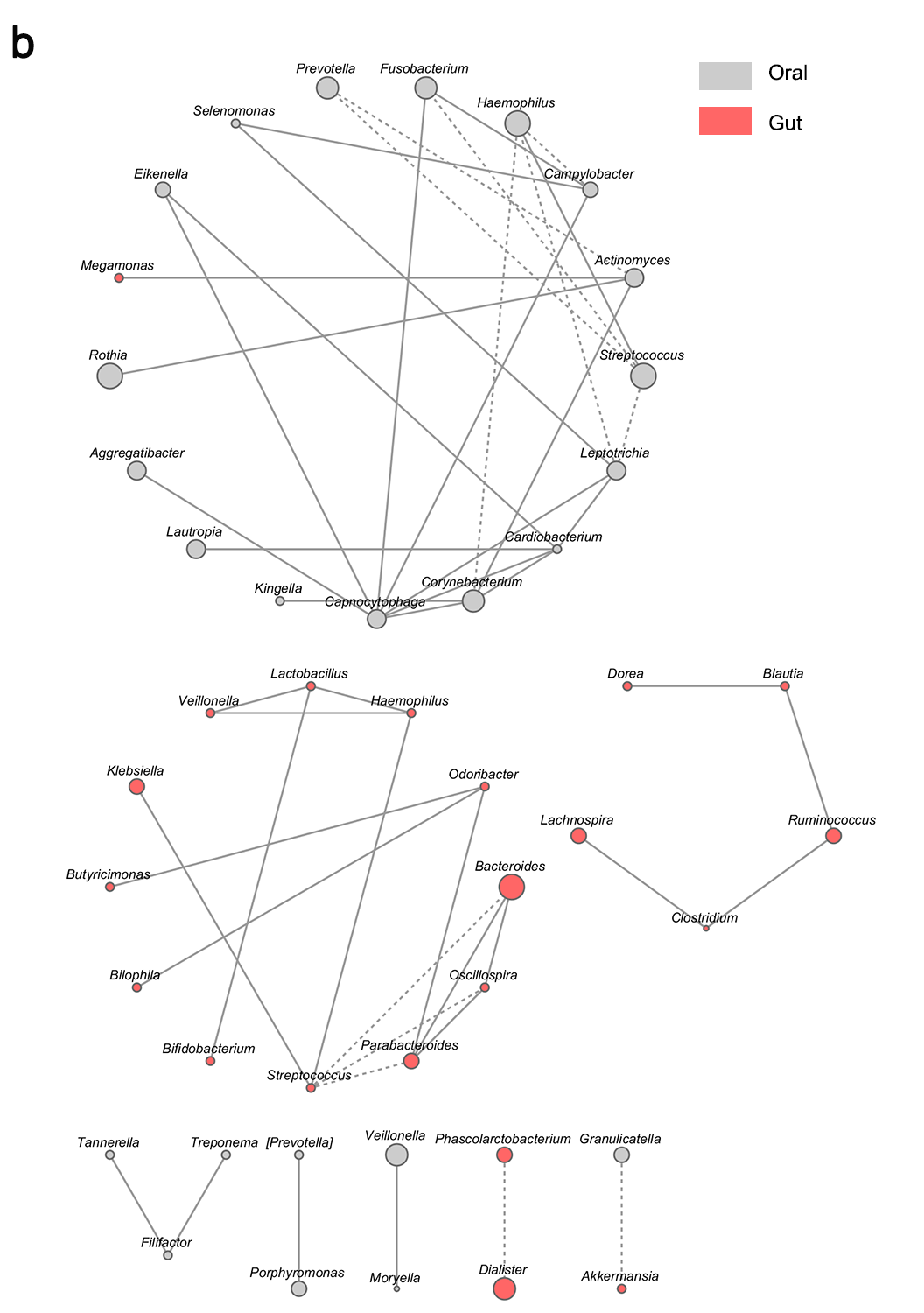


**
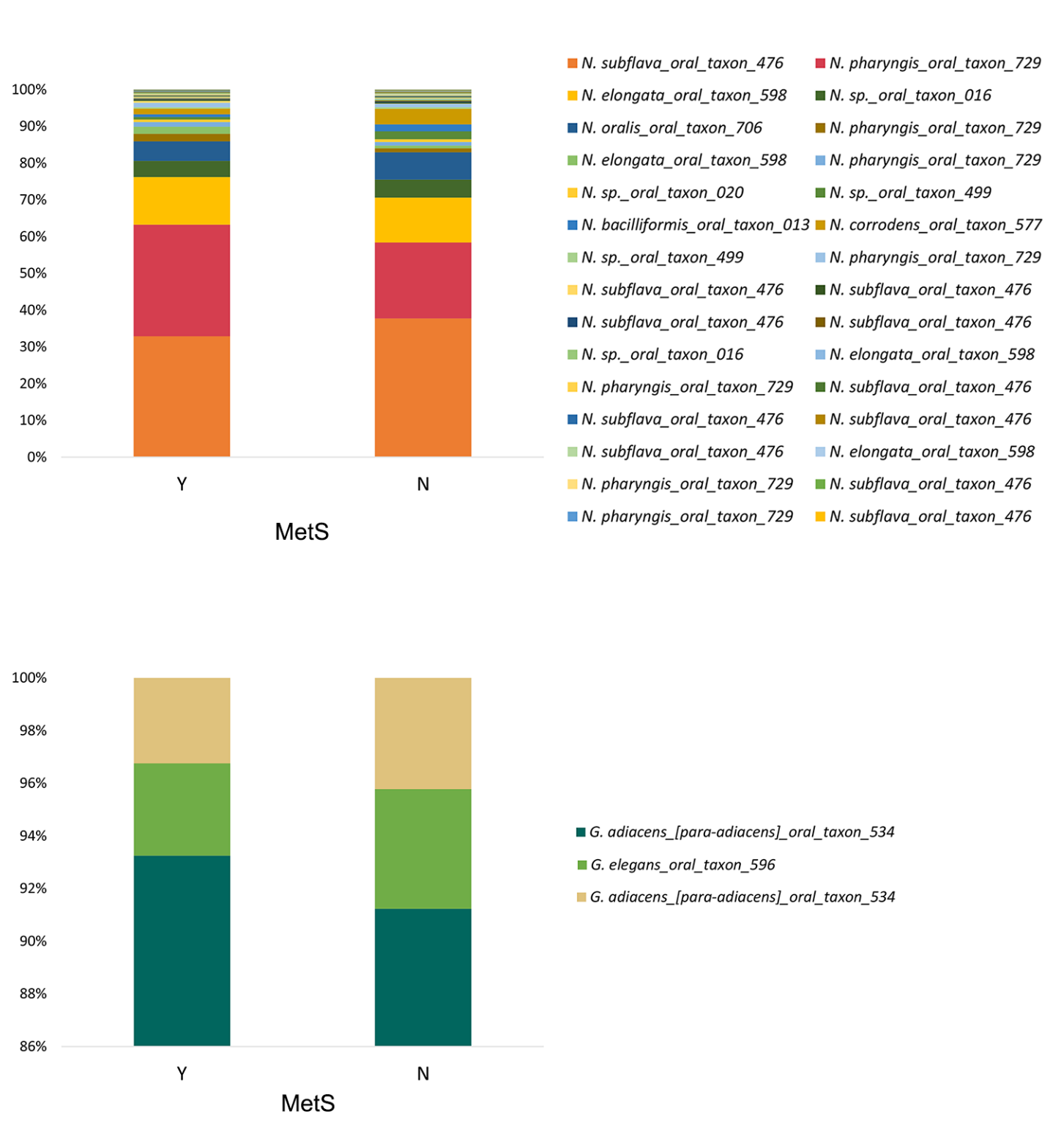
**

**Supplementary Figure 5. Taxonomic distributions of *Neisseria* and *Granulicatella* oligotypes.** Representative sequences of each oligotype were searched against the Human Oral Microbiome Database (HOMD) reference (version 13.2). A total of 46 and 3 oligotypes were identified for *Neisseria* and *Granulicatella*.

**
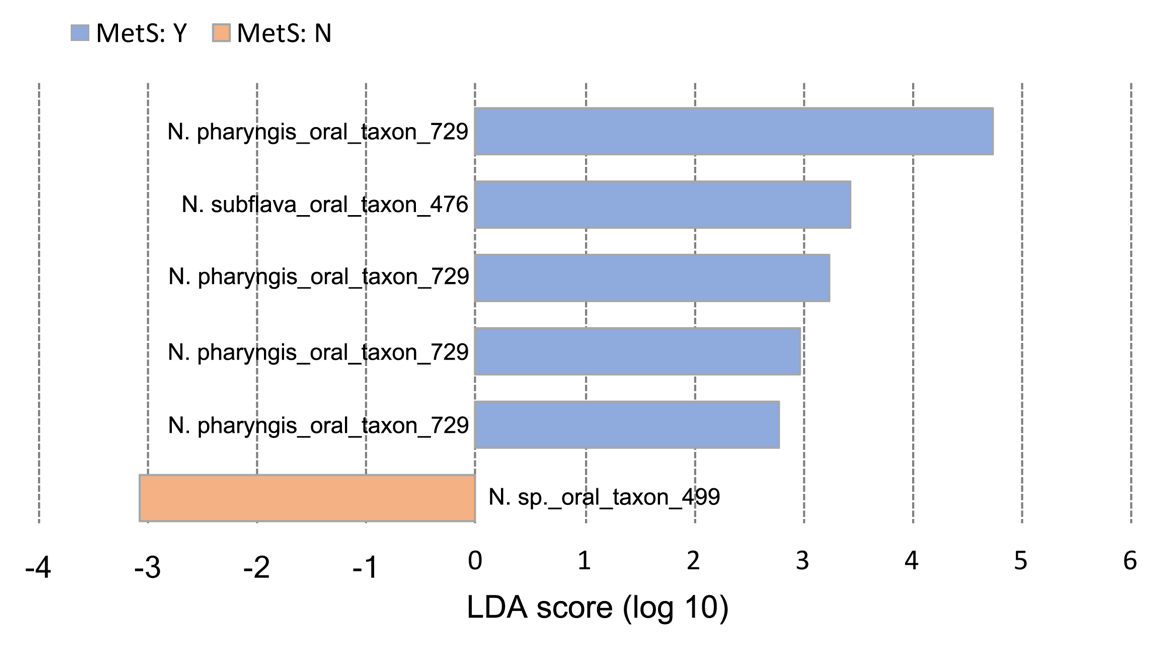
**

**Supplementary Figure 6. Comparison of *Neisseria* oligoytpes using LEfSe.**
